# Evaluating the effectiveness of adjuvant radiotherapy in addition to surgery versus surgery alone at improving oncologic outcomes for early stage buccal carcinoma: a systematic review

**DOI:** 10.1186/s40463-019-0396-x

**Published:** 2019-12-30

**Authors:** Corliss Ann Elizabeth Best, Alexandra Elizabeth Quimby, Brittany Ann Barbara Best, Dean Furgusson, Hussain Alsaffar

**Affiliations:** 1 The Department of Otolaryngology – Head and Neck Surgery, University of Ottawa, 501 Smyth Rd, Ottawa, ON K1H 8L6 Canada; 2 Ottawa Hospital Research Institute, Ottawa, Canada; 3 Department of Epidemiology and Community Medicine, University of Ottawa, Ottawa, Canada

**Keywords:** Buccal squamous cell carcinoma, Oral cavity squamous cell carcinoma, Surgery, Radiation, Early stage disease, Oncologic outcomes

## Abstract

**Objective:**

To systematically review the evidence to evaluate oncologic outcomes for patients with early stage buccal squamous cell carcinoma treated with surgery versus surgery and adjuvant radiation therapy.

**Data sources:**

Ovid MedLine, EMBASE, Google Scholar, PubMed.

**Review methods:**

The primary purpose was to perform a systematic review to determine the published literature comparing oncologic outcomes of patients with early stage (Stages I&II) buccal mucosal squamous cell carcinoma, treated with surgical resection alone versus surgery plus adjuvant radiation therapy. Oncologic outcomes of interest were overall survival, locoregional recurrence, and disease specific survival. The secondary aim was to perform a meta-analysis to quantitively compare and summarize the data on oncologic outcomes between treatments.

**Results:**

A total of 1457 studies were screened and five retrospective cohort studies (*n* = 733 patients) were eligible for quantitative analysis. Overall study quality was moderate to high. Pooled relative risk ratios using a fixed effects model did not reveal any statistically significant difference in overall survival (*p* = 0.70) or locoregional recurrence rates (*p* = 0.72) in Stage I and II disease.

**Conclusions:**

These results demonstrate there is sparse evidence comparing oncologic outcomes for early stage buccal squamous cell carcinoma treated with surgery alone versus surgery and adjuvant radiation therapy. Our findings based on a limited body of evidence suggest no obvious benefit in the addition of adjuvant radiation therapy, however robust randomized trials are warranted to reach firm conclusions.

## Introduction

Oral cavity cancer (OCCA) is a prevalent form of head and neck cancer. A particularly aggressive form of OCCA is squamous cell carcinoma (SCC) of the buccal mucosa. The buccal mucosa is defined as the mucosa lining the inner surface of the cheek and lip, extending from the line of attachment of the upper and lower alveolar ridges to the pterygomandibular raphe [1]. The incidence of buccal SCC varies globally; it accounts for the majority of oral cancers in places such as India and South America, but only 10% of all oral cavity cancer in North America and Western Europe [2, 3]. This variation may be reflective of the common risk factors, as incidence is higher in areas where chewing tobacco and betel nut chewing are common [4].

Various treatment approaches have been used to manage buccal SCC, including surgery, radiotherapy with or without chemotherapy, or all three modalities combined [2]. Despite this, buccal SCC is associated with high locoregional failure rates, even in patients with early stage disease (Stage I and II) with rates of locoregional recurrence as high as 40% [5–7]. Potential rationale for recurrence includes the intrinsically aggressive nature of the disease, and the lack of clear tissue planes within the buccal mucosa, making adequate surgical margin control difficult [8].

The typical treatment of early staged buccal SCC (Stage I and II) is surgery alone, however some institutions offer patients surgery with adjuvant radiation therapy in an attempt to improve locoregional failure rates [8]. Cancer Care Ontario recommends postoperative radiotherapy should be considered for patients with early stage SCC if there are clinical and pathological features that indicate a high risk of recurrence [9]. According to the Alberta Health Services Guidelines, if a patient has any of the following adverse risk features, recommended treatment after resection includes: extracapsular spread +/− positive margin, consider chemoradiotherapy; positive margin, consider re-resection, if not possible, consider chemoradiotherapy; perineural invasion and / or vascular embolism, consider radiotherapy alone [10]. To our knowledge, there has not yet been a systematic review performed comparing outcomes for different treatment modalities in patients with early stage buccal SCC.

The primary purpose of this study was to perform a systematic review to determine the published literature comparing oncologic outcomes of patients with early stage (Stages I and II) buccal mucosal SCC, treated with surgical resection alone versus surgery plus adjuvant radiation therapy. Oncologic outcomes of interest were overall survival (OS), locoregional recurrence (LRR), and disease specific survival (DSS). The secondary aim of this study was to perform a meta-analysis to quantitively compare and summarize the data on oncologic outcomes between these two treatments.

## Methods

We followed the Preferred Reporting Items for Systematic Review and Meta-Analyses (PRISMA) guidelines for the methodology of this systematic review and meta-analysis [11].

### Eligibility criteria

Studies were selected using Population, Intervention, Comparison, Outcome (PICO) guidelines (Table 1). We included all studies from 1947 to December 2017 published in English language in peer-reviewed journals.

**Table 1:** Inclusion and Exclusion Criteria

Population	Adult (> 18 years) patients with early stage (I, II) buccal carcinoma
Intervention	Curative intent surgery
Comparator	Surgery + adjuvant radiation
Outcome	Overall survival; disease-specific survival; locoregional recurrence
Study Design	Randomized controlled trials; retrospective and prospective cohort studies
Population	Pediatric population; non-oral cavity cancer or oral cavity subsite other than buccal mucosa; advanced stage (III, IV) buccal mucosal cancer only
Intervention	Non-curative intent surgery
Comparator	No comparator
Outcome	No oncologic outcomes
Study Design	Case series; case reports
Other	Non-English language

### Information sources and search strategy

A literature search was conducted by an experienced librarian to locate articles published between 1947 and 2017 using Ovid MedLine, EMBASE, Google Scholar and PubMed databases. The search strategy employed database-specific subject headings and keywords for “buccal mucosa”, “cheek mucosa”, “squamous cell carcinoma”, “buccal mucosa tumor/ tumour/ cancer/ neoplasm”, “surgery/ surgical excision”, “radiotherapy”, “chemotherapy”, “chemoradiotherapy”, “adjuvant/ neoadjuvant therapy”. We also performed a hand search using the abstracts from the relevant articles, which did not yield any additional articles for the analysis. See Appendix for full search strategy.

### Study selection

Studies that included adult patients (> 18 years) with early stage (I, II) buccal SCC as their population of interest, intervention of curative intent surgery, compared to surgery plus adjuvant radiation therapy, and measured overall survival, disease specific survival, and / or locoregional recurrence were included. Study designs including randomize control trials, retrospective and prospective cohort studies were included.

Studies were excluded if they included pediatric patients, non-oral cavity cancer or cancer of other oral cavity subsites, or exclusively examined outcomes in advanced stage buccal SCC. Studies that included non-curative intent surgery as the intervention, did not have a comparator group, and did not examine oncologic outcomes were also excluded. Case series of less than 10 patients, case reports designs were excluded, as well as non-English language manuscripts. See Table 1.

### Data collection and extraction

Data was extracted from the studies using a standardized and piloted data abstraction form. Titles and abstracts were independently screened by two reviewers (C.A.E.B., A.E.Q.) to assess for initial relevance. Titles or abstracts that were deemed relevant by either reviewer were obtained in full document or PDF form. Full documents were then screened to determine if they met eligibility criteria, and if so, data was extracted accordingly. Data extraction was completed by two reviewers (C.A.E.B., A.E.Q.), and included important clinical baseline variables as well as primary and secondary outcome measures (Table 2). All disagreements between reviewers were discussed and resolved by a consensus meeting including four authors (C.A.E.B., A.E.Q, B.A.B.B., H.A.).

**Table 2:** Baseline Cohort Demographics

Study name	N	Age (Median, years)	N Early Stage I + II buccal CA	N Advanced Stage (III+ IV) Buccal CA	N Surgery (Early-stage)	N Surgery + Radiation (Early-stage)	Margin Positivity	Perineural / Lymphovascular Invasion Present	Radiation Dose
Jenwitheesuk et al.	107	67	13	94	9	3	NA	NA	NA
Lin et al.	182	51	44	77	22	15	NA	NA	Median 67.2 Gy (13.2–87 Gy)
Dixit et al.	176	47	68	108	56	12	NS between groups	NA	Range 28–60 Gy
Chaudhary et al.	291	55	35	256	13	1	NA	NA	50–60 Gy
Pop et al.	38	NA	17	21	6	1	NA	NA	40–70 Gy

*CA* Cancer

*NA* Unknown

*NS* Non-significant

### Data items

Details of the extracted data are included in Table 2. Clinical variables extracted from each article included the country in which the study was performed, age of the treated patient population, total number of patients treated with early stage (I + II) tumors, number of patients treated with surgery alone and number with surgery and adjuvant radiation, radiation treatment dosage, and length of follow-up. Primary outcomes of interest included overall survival (OS), locoregional recurrence (LRR) rates, and disease-specific survival (DSS).

### Risk of Bias in individual studies

All of the studies included in our analysis were observational cohort studies. The quality of the papers was therefore assessed using the Newcastle Ottawa Scale (NOS) [10].

### Synthesis of results

Statistical analysis was completed using Stata software, version 15 (StataCorp). Continuous demographic and outcome variables were analyzed using weighted means and standard deviations. Meta-analysis using a fixed effects model was used to summarize our outcomes of interest with RRs and their associated 95% confidence intervals (CI). A RR > 1 favored surgery alone, while a RR < 1 favored surgery plus radiation therapy for the outcomes of interest. Extent and inconsistency amongst the results and the proportion of total variability accounted for by heterogeneity versus chance alone was measured with the I^2^ test and compared to the Q value.

## Results

### Study selection

**Fig. 1:**
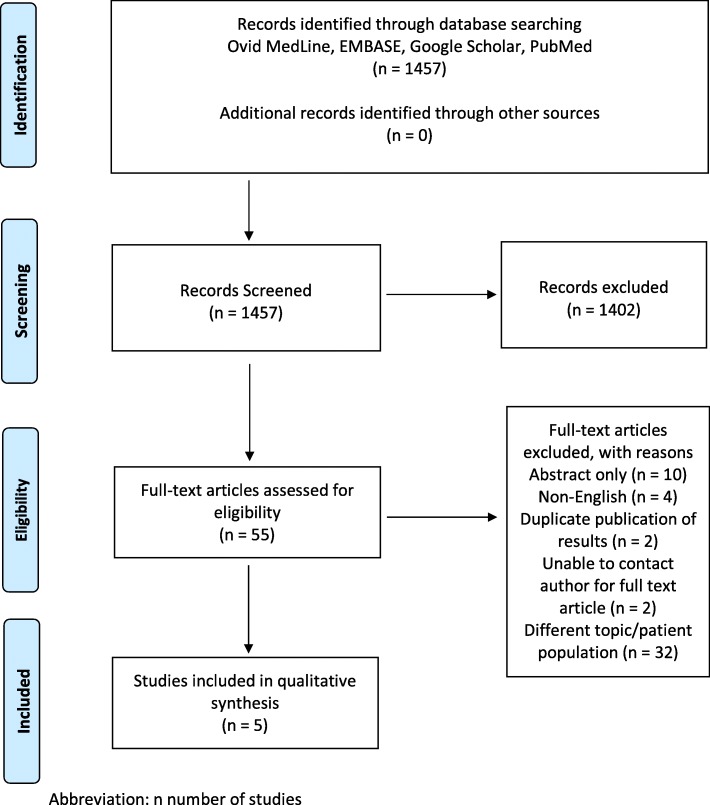
PRISMA Diagram

A total of 1457 studies were screened with 55 full text articles assessed for eligibility. Studies were excluded for the following reasons: abstract only, non-English articles, duplicate publication, unable to contact author for full text article, different topic or patient population, no comparator arm, and no oncologic outcomes. Five studies were deemed eligible for inclusion for final analysis. See Fig. 1 for search strategy and results.

### Study characteristics

The total of 733 patients were evaluated in our 5 included studies. 177 patients were classified as early stage (I and II) buccal SCC, and 556 patients were classified as advanced stage (III and IV). 106 patients with early stage buccal SCC received surgery only, and 32 patients with early stage buccal SCC received surgery and adjuvant radiation therapy. All studies were retrospective cohort studies. Baseline cohort demographics of all studies included in the review [4, 12–15] are provided in Table 2.

### Study quality assessment

Quality assessment and bias detection was assessed for all five retrospective cohort studies using the Newcastle Ottawa Scale (NOS), which assesses overall study quality on the basis of patient selection, comparability, and outcomes. The Overall Methodology and Quality scores of the 5 studies ranged from 5 to 8 stars (Maximum score = 9 stars) (Table 3).

**Table 3:** Included Study Overall Methodology and Quality Scores Using Newcastle Ottawa Scale

Study name	Selection (Maximum 4★)	Comparability (Maximum 2★)	Outcome (Maximum 3★)	Overall Methodologic Quality and Score (Maximum 9 ★)
Jenwitheesuk et al.	★★★★	★	★★	★★★★★★★ [7]
Lin et al.	★★★★	★★	★★	★★★★★★★★ [8]
Dixit et al.	★★★	★	★	★★★★★ [5]
Chaudhary et al.	★★★★	★★	★	★★★★★★★ [7]
Pop et al.	★★★★	★	★★	★★★★★★★ [7]

### Primary outcome: systematic review of existing published studies

Our research revealed that no clinical trials or prospective cohort studies have been conducted on this topic. We identified the following five retrospective cohort studies that met our inclusion criteria. A summary of included study characteristics can be found in Table 4.

**Table 4:** Included Study Characteristics

Study name	Year	Type of Study	Country	Outcome Measure	Adjusted Outcome (Y/N)	Length of follow-up	NOS Score
Jenwitheesuk et al. [12]	2010	Retrospective Cohort	Thailand	Crude OS; HR’s	N	5 years	7
Lin et al. [6]	2006	Retrospective Cohort	China	Crude OS; adjusted HR’s	Y	Median 37.1 mo (5.5–189.5)	8
Dixit et al.[13]	1998	Retrospective Cohort	India	Crude LRR; adjusted HR’s	Y	Median 32 mo (7–72)	5
Chaudhary et al. [14]	1989	Retrospective Cohort	India	Crude LRR	N	2 years	7
Pop et al.	1989	Retrospective Cohort	Netherlands	Crude LRR	N	5 years	7

*OS* Overall survival

*HR* Hazard ratio

*NOS* Newcastle-Ottawa Scale

#### Jenwitheesuk et al. (2010) [12]

Jenwitheesuk et al. published a retrospective cohort study in 2010 reviewing the clinical presentation and treatment of buccal carcinoma at their institution over a 10-year period (Khon Kaen University, Thialand; 1995–2005). They also compared survival rates across treatment modalities.

A total of 107 records of patients with newly diagnosed buccal SCC were reviewed. Thirteen patients were Stage I (*n* = 6) or II (*n* = 7). Of the patients with Stage I and II disease, 9 patients underwent surgery alone, 3 patients underwent combination surgery and radiotherapy, and 1 patient underwent radiotherapy alone.

The authors reported the 5-year overall survival rates of patients in their cohort Stage I disease as 67% and Stage II as 43%. Overall survival was also reported according to treatment group. Patients with Stage I disease who received surgery alone (*n* = 5) had a 5y survival rate of 80%; Stage I patients who received surgery plus radiation therapy (*n* = 1) had a 5y survival rate of 100%; no patient with Stage I disease received radiotherapy alone. Patients with Stage II who received surgery only (*n* = 4) had a 5y survival rate of 75%; Stage II who received surgery plus radiation therapy (*n* = 2) had a 5y survival rate of 50%; and Stage II who received radiation therapy only (*n* = 1) had a 0% 5y survival rate. Statistical analyses comparing survival rates across treatment modalities were not performed.

The authors also examined survival outcomes among patients with advanced stage disease, and their conclusions were focused on treatment recommendations for advanced stage buccal SCC. There were no specific recommendations provided concerning treatment of early stage buccal SCC.

#### Lin et al. (2006) [6]

Lin et al. retrospectively reviewed the treatment records of 121 patients diagnosed with buccal SCC over a period of 20 years (1983–2003) at their institution in Taiwan. The goals of their study were to determine objectively whether buccal cancer is a highly aggressive malignancy, and to present evidence that supports an aggressive multimodality treatment policy even for early- stage buccal cancer for which most experts currently would not recommend adjuvant therapy after surgery.

There were 44 patients classified as Stage I (*n* = 12) and Stage II (*n* = 32). Regarding patients with early-stage buccal SCC, 22 underwent surgery alone, 15 had surgery plus radiation therapy, and the remaining 7 underwent radiation therapy alone.

The 5-year OS, DSS, and locoregional control rates were provided for patients with Stage I and II disease according to treatment modality. The 5-year locoregional control rates for patients with early-stage carcinoma were 60.7% for the surgery group alone compared to 52.2% for the surgery plus radiation therapy group. Patients with early stage disease treated with radiotherapy alone had a 5-year locoregional control rate of 19.1%. The 5-year overall survival rates for patients with early stage carcinoma treated with surgery alone were 52.9, and 63.2% for the surgery plus radiation therapy group. Patients with early stage disease treated with radiotherapy alone had a 5-year overall survival of 28.6%. 5-year disease-specific survival rates for early stage disease were 62.6% for surgery alone; 63.2% for surgery plus radiotherapy; and 35.7% for radiotherapy alone. The authors performed survival analyses using Kaplan-Meier curves and the log-rank test, and found significant differences in 5-year locoregional control and overall survival in early stage buccal SCC according to treatment modality (surgery, surgery plus radiation, or radiation alone) (*p* = 0.0210 for 5-year locoregional control; *p* = 0.0489 for 5-year overall survival). There was no significant difference in 5-year disease-specific survival between treatment modalities (*p* value reported as ‘ns’). Similarly, using Cox multivariable regression analysis, they found that treatment modality was a significant prognostic factor for both overall survival and disease-specific survival.

The authors concluded that their results support the routine use of adjuvant radiation therapy for patients with early stage (T1,2N0) buccal SCC, in addition to advanced stage (Stage III + IV) disease.

#### Dixit et al. (1998) [13]

Dixit et al. conducted a retrospective cohort study of patient records from 1989 to 1993 (Gujarat Cancer and Research Institute, Ahmedabad, India) to assess the role of postoperative radiotherapy in treating carcinoma of the buccal mucosa.

A total of 68 patients were classified as having Stage I and II disease. There were 56 patients who received surgery alone (18 patients Stage I, 38 patients Stage II), and 12 patients (0 patients Stage I, 12 patients Stage II) who received surgery and adjuvant radiotherapy. Referral for post-operative radiotherapy was done at the discretion of the referring surgeon. The actuarial 3-year locoregional control rates for patients with Stage I and II disease treated with surgery alone was 71%, and Stage I and II disease treated with surgery plus radiation therapy was 75%.

The authors conducted multivariable regression analysis of factors influencing locoregional failure, including overall stage, pathologic T stage, clinical N stage, pathologic bone involvement, pathologic skin involvement, tumor grade, tumor thickness, and surgical margin status. For early stage buccal SCC (Stage I and II) a significant difference in locoregional failure rates between existed between patients who received surgery alone versus surgery plus radiation only in cases of close margins (< 2 mm) (*p* = 0.019) or increased tumor thickness (> 10 mm) (0.048).

Based on their multivariable analysis, the authors concluded that postoperative radiotherapy was effective in decreasing locoregional failure in patients with early stage buccal CA only in cases with close margins, or tumor thickness greater than 10 mm.

#### Chaudhary et al. (1989) [14]

Chaundhary et al. completed a retrospective analysis of the clinical presentation, treatment, and survival outcomes of buccal mucosal SCCs seen at the Tata Memorial Hospital, Bombay, during the year 1984. The aim of their study was to determine the relative efficacy of various treatment modalities across the four stages of buccal SCC.

Of 326 total cases analyzed, 35 patients had early stage disease (Stage I, *n* = 12; and Stage II, *n* = 27).

Of the patients with early stage disease, 13 underwent surgery alone, 1 patient had surgery with adjuvant radiation, and 21 underwent radiotherapy alone. Locoregional control rates were assessed at a follow-up duration of 2 years. Of the early stage patients who received surgery alone, 46% (*n* = 6) had good locoregional control at 2 years, 31% (*n* = 4) experienced a locoregional recurrence, and 23% (*n* = 3) were lost to follow up. Of the early stage patients who received radiotherapy alone, 48% (*n* = 10) had good locoregional control, 33% (*n* = 7) experienced locoregional recurrence, and 19% (*n* = 4) were lost to follow up. The one patient treated with combination surgery + radiation therapy was lost to follow up. No statistical analyses were performed comparing treatment success and failure rates for early stage disease across modalities.

The authors concluded that early OCCAs are equally amenable to control by surgery and radiation.

#### Pop et al. (1989) [15]

Pop et al. conducted a retrospective review of patients treated with buccal SCC at their institution (Rotterdam, Netherlands) over a 15-year period (1970–1984). Their intent was to evaluate the survival and oncologic outcomes of different treatment strategies for buccal SCC.

Of 49 total patients, 17 were classified as having early stage disease (Stage I, *n* = 5; and Stage II, *n* = 12).

Of patients with early stage disease, 6 were treated with surgery alone, and 1 was treated with surgery plus adjuvant radiotherapy. 10 patients with early stage disease were treated with radiotherapy alone. A uniform duration of follow-up was not provided by the authors. The “corrected” 5-year survival for Stage I disease was 100%, and for Stage II disease was 56%; these results were not reported according to treatment modality. Locoregional recurrence rates were reported according to stage and treatment modality: 66% of patients with early stage disease treated with primary surgery recurred locally (*n* = 2); 100% of patients with early stage disease treated with surgery plus adjuvant radiation therapy recurred locally (*n* = 1); and 30% of patients with early stage disease treated with radiation alone recurred locally (*n* = 3). Statistical analyses comparing overall survival and locoregional recurrence rates for early stage disease across treatment modalities were not performed.

The authors concluded that their results support primary radiation therapy as curative for early stage buccal SCC. They recommended a dose to the primary tumor as well as the ipsilateral N0 neck of 50Gy in 5 weeks, then continuing radiation to the primary tumor only for a total tumor dose of 70–75 Gy.

### Secondary outcome: meta-analysis of oncologic outcomes

We performed a meta-analysis to pool oncologic outcomes across our included studies as a low overall degree of heterogeneity was present across studies.

**Fig. 2:**
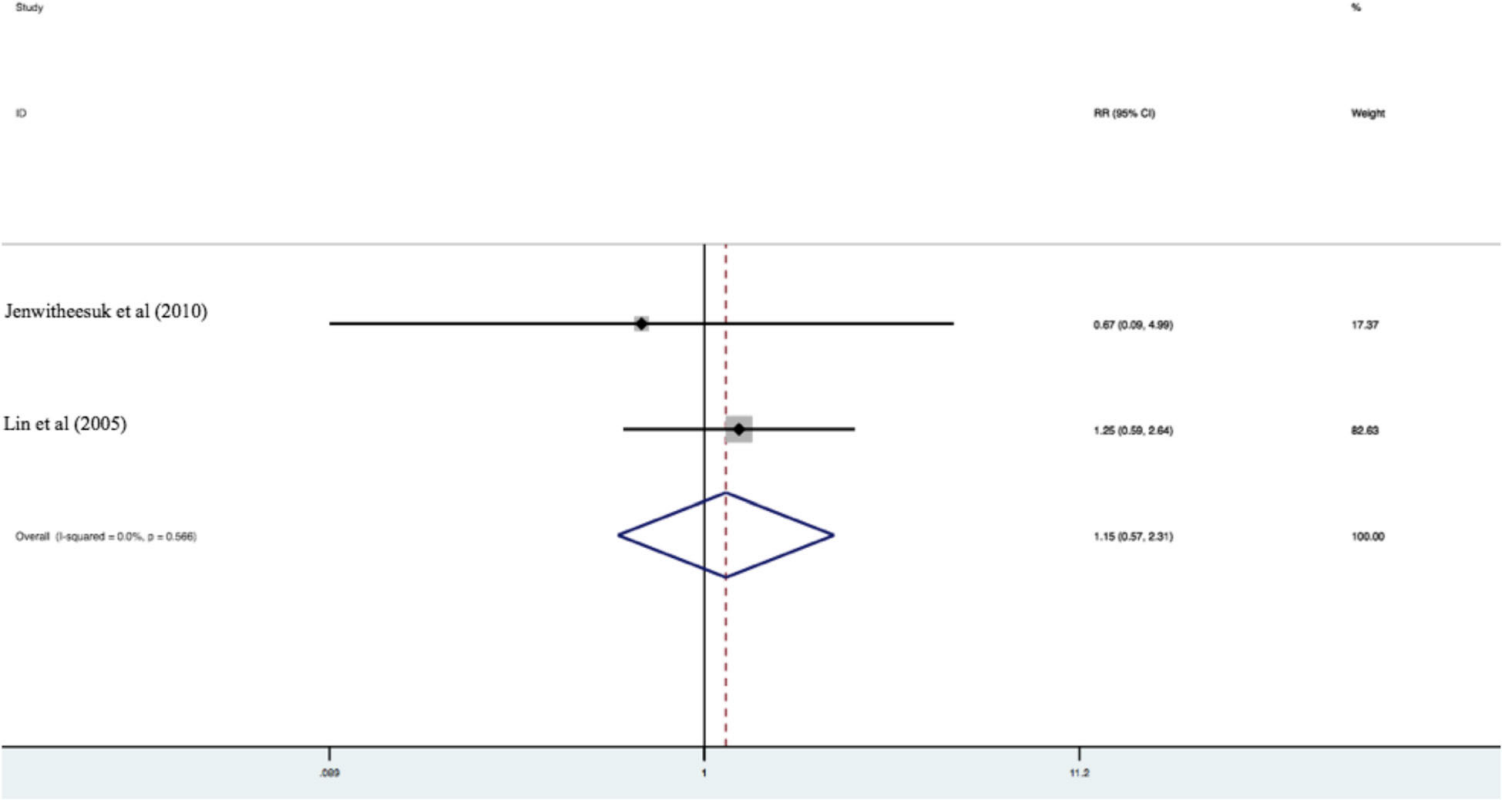
Overall survival between patients with early stage buccal SCC treated with surgery alone versus surgery and adjuvant radiation therapy (pooled RR = 1.15 [95% CI 0.57–2.31]; *p* = 0.70) (*n* = 2 included studies) [12, 13]

Risk ratios calculated using a fixed effects model revealed no statistically significant difference in overall survival between patients with early stage buccal SCC treated with surgery alone versus surgery and adjuvant radiation therapy (pooled RR = 1.15 [95% CI 0.57–2.31]; *p* = 0.70) (*n* = 2 included studies) [12, 13]. See Fig. 2.

**Fig. 3:**
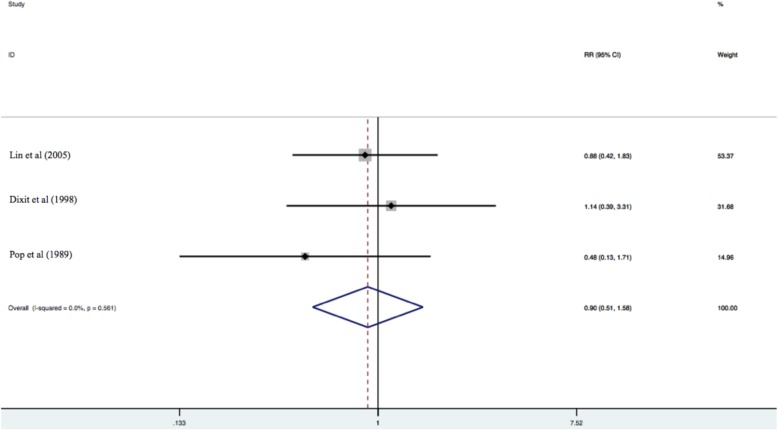
Locoregional recurrence rates among the two treatment groups (RR = 0.90 [95% CI 0.51–1.58]; *p* = 0.72) (*n* = 3 included studies) [13–15]

Similarly, there was no significant difference in locoregional recurrence rates among the two treatment groups (RR = 0.90 [95% CI 0.51–1.58]; *p* = 0.72) (*n* = 3 included studies) [13–15]. See Fig. 3.

There was insufficient data to perform meta-analysis comparing disease specific survival across treatment modalities for early stage buccal SCC.

The I^2^ statistic demonstrated that there was not significant heterogeneity in the studies included in the analysis for both OS and LRR (I^2^ = 0.0%).

## Discussion

This systematic review sought to summarize the literature examining oncologic outcomes of early stage buccal SCC treated with either surgery alone or surgery with adjuvant radiation therapy. We have demonstrated that no clinical trials or prospective cohort studies have been conducted. We did identify five retrospective cohort studies have been published on this topic in the last 30 years. To the authors’ knowledge, this has been the first systematic review that has set out to compare oncologic outcomes between these two possible treatment modalities for patients with early stage buccal mucosa carcinoma.

We believe this research question to be of importance given the high rates of locoregional failure following treatment of early stage disease. The high locoregional recurrence rate is thought to be secondary to an intrinsically aggressive nature of the disease, and to the unique buccal mucosal anatomy which lacks definitive barriers to prevent tumor spread to the buccinator muscle and adjacent buccal fat space [16]. High local recurrence rates are documented throughout the published literature, although rates vary slightly based on geographic location and study size. Sieczka et al. performed a retrospective chart review including 27 patients in New York, USA and found the local failure rate to be 40% in patients with T1-T2 buccal SCC that had been excised with negative surgical margins [5]. Similarly, a retrospective chart reviewed performed on a population out of Toronto, Canada, in 2012 reported an overall recurrence rate of 41% [2]. Lin et al. also reported local recurrence in a population in Taiwan of 41% [6]. Higher rates of locoregional recurrence come from Strome et al. in USA, who reported a 100% overall incidence of local disease recurrence in patients with stage I and II tumors treated with wide local excision alone after two years of follow up [7]. Recent literature suggests the incidence of local recurrence is slightly higher than other sites of the oral cavity [2].

As a result, some centres have advocated the routine use of post-operative radiotherapy for even early stage buccal SCC [5, 9, 13], however it has not been well-examined whether such a strategy offers patients a clear survival benefit. It is also important to highlight that radiation therapy is not without lasting adverse side effects that can severely affect patient quality of life, including xerostomia, osteoradionecrosis, pain, fatigue, and deterioration in overall function.^18^

Our systematic review has demonstrated that there are very few studies in the published literature which have directly compared oncologic outcomes between the two treatment strategies of surgery alone, or surgery with adjuvant radiation therapy, for early stage buccal SCC. Furthermore, the quality of evidence of available studies is lacking: all published studies on this topic are in the form of retrospective reviews, with no prospective or randomized trials having been completed to date. Methodologic quality of these retrospective reviews is heterogeneous, as indicated by NOS scores ranging from 5 to 8 (maximum 9); methodologic flaws include non-uniform durations of follow-up, lack of formal statistical analysis including adjustment for confounding, and a moderate degree of loss to follow-up. Additionally, an important factor in the decision to pursue adjuvant radiation after surgery is the presence of lymphovascular or perineurial invasion. Unfortunately, zero of five included studies commented on whether the tumors of patients who received adjuvant radiation had these negative prognostic features. Lastly, two of five of the included studies are outdated, and therefore raises the question whether the quality of the treatment modalities at the time of publication are still comparable to modern treatment modalities.

Based on a limited number of retrospective studies, with methodologic shortcomings as noted above, there did not appear to be any significant differences in oncologic outcomes for early stage buccal SCC (Stage I and II) treated with surgery alone compared to surgery with adjuvant radiation. However, due to the above noted limitations, the strength of conclusions allowed to be drawn from these results is limited. The authors do not think there is sufficient evidence contained herein to contribute to or modify clinical decision making in the treatment of patients with early stage buccal SCC.

## Conclusion

In summary, this is the first study that has sought out to systematically review the literature comparing oncologic outcomes between surgery alone versus surgery plus adjuvant radiation therapy for patients with Stage I and II buccal mucosal SCC (where oncologic outcomes were defined as overall survival, locoregional recurrence, and disease specific survival). We have concluded that there is a paucity of published data on this topic, with the existing literature limited to a small number of retrospective cohort studies with some inherent methodologic limitations. Bases on meta-analysis pooling of this limited amount of data, we did not find a statistically significant difference in oncologic outcomes for early stage disease when adjuvant radiation therapy was added to surgical excision alone. Importantly, we do not think there is sufficient evidence to contribute to or modify clinical decision making in the treatment of patients with early stage buccal SCC. Our study draws attention to the fact that in order to reach firm conclusions of any possible benefit of adjuvant radiation therapy for the treatment of early stage buccal SCC, further high-quality study is warranted, especially in the form of prospective randomized trial.

## Data Availability

All data gathered for the systematic review was gathered from articles cited in the paper and listed in the reference section.

## Appendix

### Search Strategy

1 (su or rt. or dt).fs. (8556363)

2 (surg* or excision or excise*).tw. (3989021)

3 resect*.tw. (720059)

4 exp. Radiotherapy/ (654618)

5 exp. antineoplastic agents/ (2856156)

6 exp. chemoradiotherapy/ or chemotherapy, adjuvant/ or neoadjuvant therapy/ or radiotherapy, adjuvant/ (137202)

7 Combined Modality Therapy/ (211830)

8 (radiotherap* or radiation therap* or chemoradiat* or chemoradiotherap* or chemotherap*).tw. (1191820)

9 or/1–8 (12800000)

10 (carcinoma/ or squamous cell Carcinoma/ or mouth neoplasms/) and bucca*.tw. (3502)

11 (bucca* mucosa* adj6 (tumor* or tumour* or cancer or neoplasm* or carcinoma* or scc)).tw. (1244)

12 (bucca* adj3 (tumor* or tumour* or cancer or neoplasm* or carcinoma* or scc)).tw. (1890).

13 10 or 11 or 12 (4472)

14 9 and 13 (2338)

15 animals/ not humans/ (5512199)

16 14 not 15 (2179)

17 16 use ppez (917) Medline

18 (cheek mucosa/ or bucca* mucosa.tw.) and squamous cell carcinoma/ (1288)

19 *mouth tumor/ and bucca*.tw. (608)

20 (bucca* mucosa* adj5 (tumor* or tumour* or cancer or neoplasm* or carcinoma* or scc)).tw. (1153)

21 (bucca* adj3 (tumor* or tumour* or cancer or neoplasm* or carcinoma* or scc)).tw. (1890)

22 or/18–21 (3302)

23 (surg* or excision or excise*).tw. (3989021)

24 resect*.tw. (720059)

25 *cancer surgery/ (28765)

26 exp. *radiotherapy/ (303894)

27 (radiotherap* or radiation therap* or chemoradiat* or chemoradiotherap* or chemotherap*).tw. (1191820)

28 exp. *antineoplastic agent/ (916291)

29 *cancer chemotherapy/ or *cancer combination chemotherapy/ (88291)

30 *adjuvant chemotherapy/ or *adjuvant therapy/ (24232)

31 *adjuvant radiotherapy/ (1784)

32 or/23–31 (5947752)

33 22 and 32 (1417)

34 (exp animal/ or nonhuman/) not exp. human/ (10525025)

35 33 not 34 (1351)

36 35 use emczd (924) Embase

37 17 or 36 (1841)

38 remove duplicates from 37 (1378)

39 38 use ppez (896) Medline

40 38 use emczd (482) Embase

## Abbreviations

DSS: Disease specific survival
LRR: Locoregional recurrence
OCCA: Oral cavity cancer
OS: Overall survival
PICO: Population Intervention Comparison Outcome
SCC: Squamous cell carcinoma
