# Correction to: Evaluating the effectiveness of adjuvant radiotherapy in addition to surgery versus surgery alone at improving oncologic outcomes for early stage buccal carcinoma: a systematic review

**DOI:** 10.1186/s40463-020-0400-5

**Published:** 2020-01-15

**Authors:** Corliss Ann Elizabeth Best, Alexandra Elizabeth Quimby, Brittany Ann Barbara Best, Dean Fergusson, Hussain Alsaffar

**Affiliations:** 1 The Department of Otolaryngology – Head sand Neck Surgery, University of Ottawa, 501 Smyth Rd, Ottawa, ON K1H 8L6 Canada; 2 Ottawa Hospital Research Institute, Ottawa, Canada; 3 Department of Epidemiology and Community Medicine, University of Ottawa, Ottawa, Canada

**Correction to: J Otolaryngol - Head Neck Surg**

**https://doi.org/10.1186/s40463-019-0396-x**

Following publication of the original article [1], the authors reported that one of the authors’ names was spelled incorrectly. In this Correction the incorrect and correct author name are shown. The original publication of this article has been corrected.

Originally the author name was published as:

- Dean Furgusson

The correct author name is:

- Dean Fergusson
